# MMGAT: a graph attention network framework for ATAC-seq motifs finding

**DOI:** 10.1186/s12859-024-05774-x

**Published:** 2024-04-20

**Authors:** Xiaotian Wu, Wenju Hou, Ziqi Zhao, Lan Huang, Nan Sheng, Qixing Yang, Shuangquan Zhang, Yan Wang

**Affiliations:** 1 Key Laboratory of Symbol Computation and Knowledge Engineering of Ministry of Education, College of Computer Science and Technology, Jilin University, Changchun, 130012 China; 2 School of Artificial Intelligence, Jilin University, Changchun, 130012 China; 3 School of Cyber Science and Engineering, Nanjing University of Science and Technology, Nanjing, 210094 China

**Keywords:** ATAC-seq, TFBSs prediction, Motif finding, Graph attention network, Coexisting probabilities

## Abstract

**Background:**

Motif finding in Assay for Transposase-Accessible Chromatin using sequencing (ATAC-seq) data is essential to reveal the intricacies of transcription factor binding sites (TFBSs) and their pivotal roles in gene regulation. Deep learning technologies including convolutional neural networks (CNNs) and graph neural networks (GNNs), have achieved success in finding ATAC-seq motifs. However, CNN-based methods are limited by the fixed width of the convolutional kernel, which makes it difficult to find multiple transcription factor binding sites with different lengths. GNN-based methods has the limitation of using the edge weight information directly, makes it difficult to aggregate the neighboring nodes' information more efficiently when representing node embedding.

**Results:**

To address this challenge, we developed a novel graph attention network framework named MMGAT, which employs an attention mechanism to adjust the attention coefficients among different nodes. And then MMGAT finds multiple ATAC-seq motifs based on the attention coefficients of sequence nodes and k-mer nodes as well as the coexisting probability of k-mers. Our approach achieved better performance on the human ATAC-seq datasets compared to existing tools, as evidenced the highest scores on the precision, recall, F1_score, ACC, AUC, and PRC metrics, as well as finding 389 higher quality motifs. To validate the performance of MMGAT in predicting TFBSs and finding motifs on more datasets, we enlarged the number of the human ATAC-seq datasets to 180 and newly integrated 80 mouse ATAC-seq datasets for multi-species experimental validation. Specifically on the mouse ATAC-seq dataset, MMGAT also achieved the highest scores on six metrics and found 356 higher-quality motifs. To facilitate researchers in utilizing MMGAT, we have also developed a user-friendly web server named MMGAT-S that hosts the MMGAT method and ATAC-seq motif finding results.

**Conclusions:**

The advanced methodology MMGAT provides a robust tool for finding ATAC-seq motifs, and the comprehensive server MMGAT-S makes a significant contribution to genomics research. The open-source code of MMGAT can be found at https://github.com/xiaotianr/MMGAT, and MMGAT-S is freely available at https://www.mmgraphws.com/MMGAT-S/.

**Supplementary Information:**

The online version contains supplementary material available at 10.1186/s12859-024-05774-x.

## Introduction

Transcription factors (TFs) and their binding sites not only play important roles in orchestrating a variety of biological processes, but have also emerged as critical contributors to the development of diseases, highlighting their significance in understanding gene regulation [1]. DNA motifs are a set of specific binding sequences of a TF, characterized by a recurring pattern known as its motif pattern, which reflects the TF’s binding preferences and specificity [2]. Motif finding aims to find conserved transcription factors binding sites (TFBSs) from high-throughput sequencing data, such as Assay for Transposase-Accessible Chromatin using sequencing (ATAC-seq) data [3]. ATAC-seq is used to investigate genome-wide chromatin accessibility by inserting Tn5 transposase into open chromatin regions to generate DNA fragments suitable for sequencing [4]. The binding of TFs to DNA sequences prevents the Tn5 transposase from cleaving the DNA sequences, creating a protective region known as an ATAC-seq footprint [5]. By detecting these footprints, multiple TF regions bound to the genome can be found from the ATAC-seq dataset. Because ATAC-seq has access to all open regions of the genome, it is convenient for TFBSs prediction and motifs finding.

Various methods have been devised to find ATAC-seq motifs. Traditional motif finding approaches primarily rely on statistical methods [6]. Statistical methods such as TOBIAS and TRACE employ known motif databases to scan sequences, and identify DNA sequences that meet specific criteria as TFBSs [7, 8]. However, these methods tend to be inefficient when applied to massive datasets and are limited by the available motif databases. This may result in the omission of novel motifs that have not yet been cataloged. With the advancement of deep learning technology, convolutional neural network (CNN)-based methods for motif finding have emerged [9]. FactorNet and scFAN utilize a convolutional kernel to detect specific motifs in sequences using CNNs [10, 11]. However, these methods are limited by their dependence on the kernel width, which leads to finding some motifs with fixed length. In recent years, graph neural networks (GNNs) have been applied to bioinformatics applications such as protein–protein interaction prediction and genomic sequence analysis [12]. MMGraph is an important study introducing GNNs to find ATAC-seq motifs, which achieved remarkable performance [13]. However, the limitation of MMGraph is the direct application of edge weights between nodes, which restricts its capacity to assess the significance of adjacent nodes for a specific target node. This constraint hampers its effectiveness in leveraging edge weight information to a more optimal extent.

**Fig. 1:**
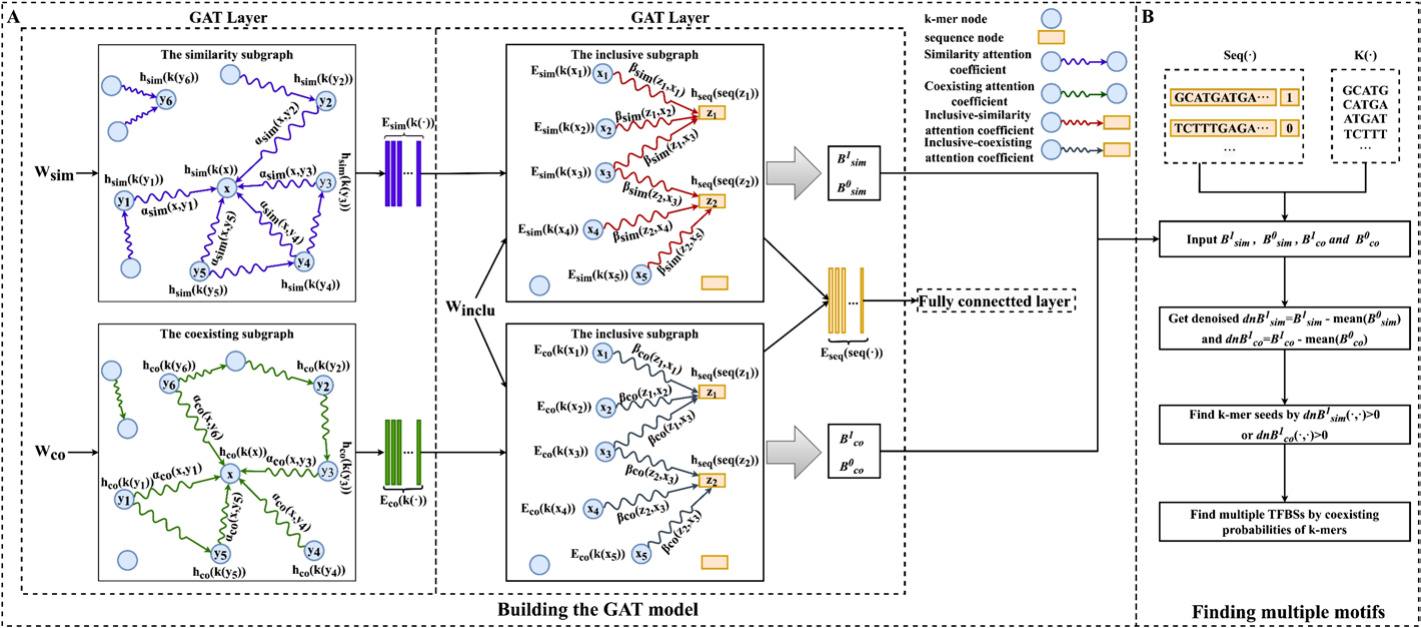
MMGAT method. (A) The first layer of MMGAT initializes embeddings ${h}_{sim}\left(k\left(\cdot \right)\right)$ and ${h}_{co}\left(k\left(\cdot \right)\right)$ for k-mer nodes ${\text{k}}\left(\cdot \right)$ in similarity and coexisting subgraphs, respectively. It employs an attention mechanism to independently learn k-mer embeddings ${E}_{sim}\left(k\left(\cdot \right)\right)$ and ${E}_{co}\left(k\left(\cdot \right)\right)$ in both subgraphs. Subsequently these two kinds of k-mer embeddings are input to the second layer to learn inclusive-similarity and inclusive-coexisting attention coefficients in inclusive subgraphs respectively. Then these two types of attention coefficients and two types of k-mer embeddings are aggregated as the embedding of sequence nodes. Finally, the sequence embeddings are input to the fully connected layer to predict TFBSs. (B) MMGAT finds k-mer seeds based on inclusive-similarity and inclusive-coexisting attention coefficients learned in the second layer, and then finds TFBSs of multiple lengths based on coexisting probabilities

To address this limitation, we propose a novel graph attention network (GAT) framework named MMGAT for TFBS prediction and ATAC-seq motif finding (Fig. 1). The first layer of MMGAT employs the attention mechanism to discriminate the relative importance of weights between k-mer nodes and thus adjusts their attention coefficients to learn k-mer node embeddings. The second layer uses the GAT to distinguish the attention coefficients between different k-mer nodes and the target sequence node, and aggregates the k-mer node embeddings to represent the sequence embeddings. The last layer is a fully connected neural network for predicting TFBSs. In addition, the MMGAT framework utilizes model-learned attention coefficients and coexisting probabilities of k-mers to find multiple motifs. We validate the performance of MMGAT using 180 human and 80 mouse ATAC-seq datasets from the ENCODE project [14]. Our method outperformed existing models in predicting TFBSs, achieving the highest average precision, recall, F1_score, ACC, AUC, and PRC scores on human and mouse ATAC-seq datasets. Additionally, MMGAT found 389 and 356 higher quality motifs than existing models in these respective datasets. Considering the notable success achieved by MMGAT and the scarcity of dedicated servers for ATAC-seq motif finding, we developed MMGAT-S. This public web server hosts the MMGAT model, the MMGraph model and others. With this implementation, configuring environments is not necessary, and users can effortlessly process ATAC-seq data without any programming knowledge. MMGAT-S visualizes the motif finding results of MMGAT in the form of motif logos and position probability matrices (PPMs). Additionally, MMGAT-S integrates several existing tools, such as AME, which allows for motif enrichment analysis of found TFBSs, and GOMo, which can perform Gene Ontology (GO) enrichment analysis of found motifs [15, 16].

## Methods

### Original MMGraph method

In our previous work, we proposed the MMGraph, which is primarily built on the GNN and employs k-mers coexisting probabilities to find multiple ATAC-seq motifs [13]. The methodology of MMGraph includes three main components.

#### Constructing the heterogeneous graph

It involves labeling sequences $seq\left(\cdot \right)$ with positive or negative markers based on the presence of TFBSs and trimming them into k-mers $k\left(\cdot \right)$ with $lenk=length\left(k\left(\cdot \right)\right)$. These n sequences $seq\left(\cdot \right)$ and m k-mers $k\left(\cdot \right)$, are two types of nodes in a heterogeneous graph. This heterogeneous graph encompasses three types of edges: similarity edges, coexisting edges, and inclusive edges.

#### Building the GNN model

MMGraph divides the heterogeneous graph into multiple subgraphs based on three edge types to deal with the relationships between different nodes separately. In the similarity subgraph, the weight of similarity edges between the m k-mer nodes form a $m\times m$ similarity subgraph weight matrix, denoted as ${W}_{sim}$. Similarly, in the coexisting subgraph, the weight of coexisting edges between the m k-mer nodes form a $m\times m$ coexisting subgraph weight matrix, denoted as ${W}_{co}$. In particular, ${W}_{sim}$ and ${W}_{co}$ are both symmetric matrices. And in the inclusive subgraph, the inclusive edges between the m k-mer nodes and n sequence nodes form an $m\times n$ inclusive subgraph weight matrix, denoted as ${W}_{inclu}$. Then the GNN model is trained based on these subgraphs, where the first layer learns the embedding of k-mer, the second layer learns the embedding of sequences, and the third layer predicts the TFBSs using a fully connected layer.

#### Finding multiple motifs

This is achieved by calculating mutual information (MI) between k-mers and sequences based on their embeddings. The process includes steps like generating and denoising MI matrices, identifying the k-mer in the positive sequences that satisfies the denoised mutual information value greater than 0 as the k-mer seeds $kseed\left(\cdot \right)$, and merging them based on their coexisting probabilities to find candidate TFBSs. Finally, MMGraph finds multiple TFBSs of different lengths by merging overlapping candidate TFBSs.

### MMGAT method

MMGAT mainly improves the components of MMGraph for building the GNN models and finding multiple motifs. MMGAT updates the first and second layers of the GNN model by introducing an attention mechanism to learn k-mer and sequence embeddings, while the third layer still uses the fully connected layer to predict TFBSs (Fig. 1A). MMGAT replaces MI by using the attention coefficients between sequence nodes and k-mer nodes to find k-mer seeds in finding multiple motifs process (Fig. 1B).

#### The first layer of MMGAT

A graph attention mechanism is employed in both the similarity and coexisting subgraphs to learn the embeddings of k-mers $k\left(\cdot \right)$ (Fig. 1A). These embeddings are respectively denoted as ${E}_{sim}\left(k\left(\cdot \right)\right)\in {\mathbb{R}}^{{d}_{k}\times 1}$ and ${E}_{co}\left(k\left(\cdot \right)\right)\in {\mathbb{R}}^{{d}_{k}\times 1}$, where ${d}_{k}$ represent the embedding dimensions for the k-mer $k\left(\cdot \right)$. The similarity subgraph weight matrix ${W}_{sim}$ and the coexisting subgraph weight matrix ${W}_{co}$ are normalized as showing in Eqs. 1 and 2, serving as the initial embedding for the k-mer node $k\left(\cdot \right)$.

1 $${h}_{sim}\left(k\left(x\right)\right)=\frac{{W}_{sim}\left(:,x\right)}{{\sum }_{y\in {\mathcal{N}}_{sim}\left(x\right)}{W}_{sim}\left(x,y\right)}$$

2 $${h}_{co}\left(k\left(x\right)\right)=\frac{{W}_{co}\left(:,x\right)}{{\sum }_{y\in {\mathcal{N}}_{co}\left(x\right)}{W}_{co}\left(x,y\right)}$$

where ${h}_{sim}\left(k\left(x\right)\right)\in {\mathbb{R}}^{{\text{m}}\times 1}$ and ${h}_{co}\left(k\left(x\right)\right)\in {\mathbb{R}}^{{\text{m}}\times 1}$ represent the initial embedding of $k\left(x\right)$ based on the similarity subgraph and the coexisting subgraph, respectively. Additionally, ${\mathcal{N}}_{sim}\left(x\right)$ refers to the set of neighbor nodes of the k-mer node $k\left(x\right)$ in the similarity subgraph, and ${\mathcal{N}}_{co}\left(x\right)$ refers to the set of neighbor nodes of $k\left(x\right)$ in the coexisting subgraph.

MMGAT calculates the attention scores ${e}_{sim}\left(x,y\right)$ and ${e}_{co}\left(x,y\right)$ for k-mer node $k\left(x\right)$ and its neighboring node $k\left(y\right)$ in the similarity and coexisting subgraphs, respectively, according to Eqs. 3 and 4.

3 $${e}_{sim}\left(x,y\right)=\sigma \left({a}_{sim}^{\mathrm{\rm T}}\cdot \left[{W}^{sim}{h}_{sim}\left(k\left(x\right)\right)\Vert {W}^{sim}{h}_{sim}\left(k\left(y\right)\right)\right]\right), y\in {\mathcal{N}}_{sim}\left(x\right)$$

4 $${e}_{co}\left(x,y\right)=\sigma \left({a}_{co}^{\mathrm{\rm T}}\cdot \left[{W}^{co}{h}_{co}\left(k\left(x\right)\right)\Vert {W}^{co}{h}_{co}\left(k\left(y\right)\right)\right]\right), y\in {\mathcal{N}}_{co}\left(x\right)$$

where ${a}_{sim}$ and ${a}_{co}$ represent the attention vectors for the similarity subgraph and the coexisting subgraph, respectively, and their dimensions are both ${2d}_{k}\times 1$. Similarly, ${W}^{sim}$ and ${W}^{co}$ denote the shared weight matrices for the similarity and coexisting subgraphs, and their dimensions are both ${d}_{k}\times m$. The $||$ represents concatenation operation, and $\sigma \left(\cdot \right)$ denotes the activation function, which is LeakyReLU in this study.

The attention scores ${e}_{sim}\left(x,y\right)$ and ${e}_{co}\left(x,y\right)$ are normalized using the softmax function, as outlined in Eqs. 5 and 6. This step yields the similarity attention coefficients ${\alpha }_{sim}\left(x,y\right)$ and the coexisting attention coefficients ${\alpha }_{co}\left(x,y\right)$.

5 $${\alpha }_{sim}\left(x,y\right)=\frac{exp\left({e}_{sim}\left(x,y\right)\right)}{{\sum }_{p\in {\mathcal{N}}_{sim}\left(x\right)}exp\left({e}_{sim}\left(x,p\right)\right)}$$

6 $${\alpha }_{co}\left(x,y\right)=\frac{exp\left({e}_{co}\left(x,y\right)\right)}{{\sum }_{p\in {\mathcal{N}}_{co}\left(x\right)}exp\left({e}_{co}\left(x,p\right)\right)}$$

Then the embeddings ${E}_{sim}\left(k\left(\cdot \right)\right)$ and ${E}_{co}\left(k\left(\cdot \right)\right)$ of k-mer node $k\left(x\right)$ in the similarity and coexisting subgraphs can be calculated utilizing Eqs. 7 and 8, based on the embeddings of adjacent nodes and their corresponding attention coefficients.

7 $${E}_{sim}\left(k\left(x\right)\right)=ReLU\left({\sum }_{y\in {\mathcal{N}}_{sim}}{\alpha }_{sim}\left(x,y\right){W}^{sim}{h}_{sim}\left(k\left(y\right)\right)\right)$$

8 $${E}_{co}\left(k\left(x\right)\right)=ReLU\left({\sum }_{y\in {\mathcal{N}}_{co}}{\alpha }_{co}\left(x,y\right){W}^{co}{h}_{co}\left(k\left(y\right)\right)\right)$$

where ReLU represents the rectified linear unit function.

#### The second layer of MMGAT

MMGAT employs GAT to learn the embedding of the sequence $seq\left(\cdot \right)$ as ${E}_{seq}\left(seq\left(\cdot \right)\right)\in {\mathbb{R}}^{{d}_{seq}\times 1}$ in the inclusive subgraph (Fig. 1A), where ${d}_{seq}$ denotes the embedding dimension of the sequence node $seq\left(\cdot \right)$ and is equal to ${d}_{k}$. The embedding of neighbor k-mer nodes learned in different subgraphs will show different importance in learning sequence node embedding. We apply the attention mechanism to learn the importance of neighbor k-mer node embeddings ${E}_{sim}\left(k\left(\cdot \right)\right)$ and ${E}_{co}\left(k\left(\cdot \right)\right)$ to the target sequence node respectively, and aggregate the feature information of these neighbor nodes to form the sequence node embedding. We use the feature transformation matrix ${W}^{inclu}$ to project the inclusive subgraph weight matrix ${W}_{inclu}$ into the feature space of dimension ${d}_{seq}$, which serves as the initialized embedding of the sequence nodes. Here the dimension of ${W}^{inclu}$ is $m\times {d}_{seq}$. This process is shown in Eq. 9.

9 $${h}_{seq}\left(seq\left(z\right)\right)={{W}_{inclu}\left(:,z\right)}^{\mathrm{\rm T}}\times {W}^{inclu}$$

where ${{\varvec{h}}}_{{\varvec{s}}{\varvec{e}}{\varvec{q}}}\left({\varvec{s}}{\varvec{e}}{\varvec{q}}\left({\varvec{z}}\right)\right)\in {\mathbb{R}}^{{1\times d}_{seq}}$ represents the initial embedding of sequence ${\varvec{s}}{\varvec{e}}{\varvec{q}}\left({\varvec{z}}\right)$.

Then we compute the attention scores ${b}_{sim}\left(z,x\right)$ and ${b}_{co}\left(z,x\right)$ of neighboring k-mer node $k\left(x\right)$ to the target sequence node $seq\left({\text{z}}\right)$ based on the k-mer node embeddings ${E}_{sim}\left(k\left(\cdot \right)\right)$ and ${E}_{co}\left(k\left(\cdot \right)\right)$, respectively, in the inclusive subgraph according to Eqs. 10 and 11.

10 $${b}_{sim}\left(z,x\right)=\sigma \left({h}_{seq}\left(seq\left(z\right)\right)\times {E}_{sim}\left(k\left(x\right)\right)\right), x\in {\mathcal{N}}_{inclu}\left(z\right)$$

11 $${b}_{co}\left(z,x\right)=\sigma \left({h}_{seq}\left(seq\left(z\right)\right)\times {E}_{co}\left(k\left(x\right)\right)\right) , x\in {\mathcal{N}}_{inclu}\left(z\right)$$

where ${\mathcal{N}}_{inclu}\left(z\right)$ refers to the set of neighbor nodes of the sequence node $seq\left({\text{z}}\right)$ in the inclusive subgraph.

The attention scores ${b}_{sim}\left(z,x\right)$ and ${b}_{co}\left(z,x\right)$ are normalized using the softmax function to obtain the inclusive-similarity attention coefficient ${\beta }_{sim}\left(z,x\right)$ and the inclusive-coexisting attention coefficient ${\beta }_{co}\left(z,x\right)$, as described in Eqs. 12 and 13. These attention coefficients ${\beta }_{sim}$ and ${\beta }_{co}$ are used to construct four attention matrices ${\mathcal{B}}_{sim}^{1}$, ${\mathcal{B}}_{sim}^{0}$, ${\mathcal{B}}_{co}^{1}$ and ${\mathcal{B}}_{co}^{0}$ according to the labels positive and negative of the sequence.

12 $${\beta }_{sim}\left(z,x\right)=\frac{exp\left({b}_{sim}\left(z,x\right)\right)}{{\sum }_{q\in {\mathcal{N}}_{inclu}\left(z\right)}exp\left({b}_{sim}\left(z,q\right)\right)}$$

13 $${\beta }_{co}\left(z,x\right)=\frac{exp\left({b}_{co}\left(z,x\right)\right)}{{\sum }_{q\in {\mathcal{N}}_{inclu}\left(z\right)}exp\left({b}_{co}\left(z,q\right)\right)}$$

Finally, MMGAT computes sequence node embedding by aggregating these neighboring k-mer node embeddings and their attention coefficients based on Eq. 14.

14 $${E}_{seq}\left(seq\left(z\right)\right)=ReLU\left({\sum }_{x\in {\mathcal{N}}_{inclu}\left(z\right)}\left({\beta }_{sim}\left(z,x\right){E}_{sim}\left(k\left(x\right)\right)+{\beta }_{co}\left(z,x\right){E}_{co}\left(k\left(x\right)\right)\right)\right)$$

#### Finding multiple motifs

Both inclusive-similarity and inclusive-coexisting attention coefficients between a sequence node and a k-mer node indicates the importance of that k-mer node to the sequence node. We consider the average values $mean\left({\mathcal{B}}_{sim}^{0}\right)$ and $mean\left({\mathcal{B}}_{co}^{0}\right)$ of the attention coefficient matrices of the negative sequences as background noise (Fig. 1B). Then we compute the denoised attention coefficient matrices $dn{\mathcal{B}}_{sim}^{1}={\mathcal{B}}_{sim}^{1}-mean\left({\mathcal{B}}_{sim}^{0}\right)$ and $dn{\mathcal{B}}_{co}^{1}={\mathcal{B}}_{co}^{1}-mean\left({\mathcal{B}}_{co}^{0}\right)$. For each k-mer $k\left(x\right)$ on each positive sequence $seq\left({\text{z}}\right)$, we define the k-mer $k\left(x\right)$ as a k-mer seed $kseed\left(x\right)$ as long as it satisfies $dn{\mathcal{B}}_{sim}^{1}\left(z,x\right)>0 or dn{\mathcal{B}}_{co}^{1}\left(z,x\right)>0$. For each k-mer seed $kseed\left(x\right)$, we use a strategy similar to MMGraph to find multiple TFBSs with different lengths [13].

## Experiment and results

### Data collection and processing

The ENCODE project provides detailed annotations of high-throughput sequencing results, offering various types of data files [14]. Among these, the Browser Extensible Data NarrowPeak (bed narrowPeak) file contains information about genomic regions identified through high-throughput sequencing techniques, such as their chromosomal location, start and end positions, statistical significance, peak intensity, and other relevant details. The Binary Alignment/Map (bam) file, on the other hand, is a binary format storing genomic sequence data, including alignments of sequence reads to a reference genome, along with quality scores and additional metadata. We consider a pair of ATAC-seq data’s bed and bam files as an ATAC-seq dataset. For our analysis, we have downloaded 180 human ATAC-seq datasets (Additional file 1: Table S1) and 80 mouse ATAC-seq datasets (Additional file 1: Table S2) from the ENCODE project.

For each ATAC-seq dataset, we initially detect footprints within the data using existing tools like TOBIAS and HINT-ATAC [7, 17]. TOBIAS not only detects footprints but also assigns a score to each, based on a single-base resolution characterization of the footprint pattern. This scoring reflects the accessibility and depth of the footprint, providing crucial information about whether a transcription factor is bound at that site. We then intersect the top-1500 ranked footprints identified by TOBIAS with those found by HINT-ATAC. The rationale for ranking footprints based on TOBIAS scores lies in the improved accuracy of distinguishing between bound and unbound sites, thereby offering more reliable footprint data for our research. We next employ bedtools to trim the intersected footprints from their centers, thereby generating sequences, denoted as $seq\left(\cdot \right)$, each extending to 101 base pairs (bp) [18]. These sequences are identified as positive and are assigned a label of ‘1’. Then we shuffle the nucleotides within each positive sequence to generate corresponding negative sequences, which are then labeled ‘0’. This approach results in a sequence set $Seq$, containing n sequences.

For each dataset, we allocated 80% of the $Seq$ as training data, 10% as validation data, and 10% as test data. Subsequently, using a sliding window of size $lenk$ and a step size of one base, we split sequence $seq\left(\cdot \right)$ into k-mers $k\left(\cdot \right)$, resulting in a collection of k-mers ${K}_{seq}\left(\cdot \right)$. This process was repeated for every sequence in $Seq$, resulting in a k-mer set $K$ containing $m$ unique k-mers.

### Experiment settings

We trained the MMGAT model using the Adam optimizer for 300 epochs, setting the initial learning rate at 0.02 with a natural decay rate of 0.001 [19, 20]. To prevent overfitting, a dropout rate of 0.3 was employed. The $lenk$ was set to 5. In our experiments, the dimensions ${d}_{k}$ and ${d}_{seq}$ were both set to 100 as the optimal parameters for the MMGAT model through experiments on 20 validation sets of human ATAC-seq data. Existing ATAC-seq motif finding methods scFAN, FactorNet, and MMGraph were used as comparison models [10, 11, 13]. Precision, recall, F1_score, ACC, AUC, and PRC were used to assess the ability of the models to predict TFBSs. To evaluate the quality of the motifs found by the models, we used the TOMTOM tool and the HOCOMOCO motif database to match the *p* value, E-value and q-value of the found motifs [21, 22]. In addition, the scalability score was used in this study to measure the running efficiency of the various models [23]. In this case, a higher scalability score indicates a more efficient operation of the model.

### Results of TFBSs prediction

TFBSs prediction aims to predict whether the input sequence contains TFBSs, which is a binary classification task. We conducted TFBS predictions on 180 human ATAC-seq datasets and 80 mouse ATAC-seq datasets, evaluating model performance using six metrics. Furthermore, in order to deeply investigate how the changes of the first and second layers in the MMGAT model affect the TFBSs prediction performance, we designed and implemented two comparison experiments. These two comparison experiments, named “MMGraph+GL1” and “MMGraph+GL2”, were used to evaluate the effects of introducing changes in the first and second layers of the graph structure on the prediction results, respectively. As shown in Table 1 and Additional file 1: Table S3, MMGAT achieved the highest average scores for all six metrics on human and mouse ATAC-seq datasets. Specifically, on the human datasets, the scores were 0.925, 0.921, 0.920, 0.921, 0.970, and 0.965, respectively, while on the mouse datasets, our model obtained 0.893, 0.884, 0.883, 0.884, 0.952, and 0.953, respectively. In particular, MMGAT increased recall by 2.56% and 6.51% on the human and mouse ATAC-seq datasets, respectively, compared to these comparison models. Our analysis of the standard deviation across these metrics reveals that MMGAT exhibits the smallest standard deviation. This observation underscores that MMGAT not only achieves superior prediction performance but also demonstrates exceptional stability in its predictions. Taking the GSE172538 dataset as an example, Figure S1 shown ROC curves of six models, among which MMGAT achieved the highest AUC scores. Notably, models employing GNNs have shown superior performance compared to those utilizing CNNs. Among these, our GAT model MMGAT outperforms the GNN model MMGraph. This underlines the efficacy of MMGAT in predicting TFBSs on ATAC-seq data. Moreover, it demonstrates MMGAT’s capability in representing the embeddings of both k-mer and sequence nodes. In addition, “MMGraph + GL1” and “MMGraph + GL2” show performance improvements in most evaluation metrics compared to MMGraph, however, these improvements still fall short of the performance of MMGAT.

**Table 1:** Mean and standard deviation of precision, recall, F1_score, ACC, AUC, and PRC scores of six models on 180 human ATAC-seq datasets

Models	scFAN	Factornet	MMGraph	MMGraph + GL1	MMGraph + GL2	MMGAT
Precision	0.793 ± 0.066	0.801 ± 0.068	0.907 ± 0.037	0.911 ± 0.036	0.910 ± 0.030	**0.925 ± 0.020**
Recall	0.748 ± 0.093	0.781 ± 0.078	0.898 ± 0.043	0.910 ± 0.037	0.902 ± 0.036	**0.921 ± 0.022**
F1_score	0.732 ± 0.117	0.775 ± 0.084	0.897 ± 0.044	0.909 ± 0.037	0.901 ± 0.037	**0.920 ± 0.022**
ACC	0.748 ± 0.093	0.781 ± 0.078	0.898 ± 0.043	0.909 ± 0.037	0.901 ± 0.036	**0.921 ± 0.022**
AUC	0.874 ± 0.094	0.876 ± 0.070	0.962 ± 0.023	0.964 ± 0.021	0.963 ± 0.021	**0.970 ± 0.017**
PRC	0.878 ± 0.088	0.875 ± 0.072	0.956 ± 0.027	0.957 ± 0.028	0.957 ± 0.027	**0.965 ± 0.022**

The bold black in table represents the highest value

### Results of ATAC-seq motifs finding

Motif finding is the process of extracting multiple ATAC motifs from input sequences. In addition to the comparison experiments with scFAN, FactorNet and MMGraph, we added an ablation experiment. We only improved the GNN model of MMGraph to GAT model, still using MI and coexisting probabilities of k-mers to find motifs, and then denote this ablation experiment using 'MMGraph + GAT'. This ablation experiment is used to examine the efficacy of finding motifs using the attention coefficient and coexisting probabilities of k-mers. It is worth mentioning that this ablation experiment was only used for the ATAC-seq motif finding task since 'MMGraph + GAT' performed consistently with MMGAT in the TFBSs prediction task. We used motifs number to assess the models' ability to find more motifs and *p value* to assess the models' ability to find higher quality motifs. The *p* value is calculated by comparing the match score of a found motif to the probability that it can be expected to receive that score when a motif is randomly generated. We consider a motif with a *p* value less than 0.05, i.e. $-{{\text{log}}}_{10}\left(p\_value\right)>-{{\text{log}}}_{10}\left(0.05\right)=1.301$, to be a significant motif. The E-value quantifies the expected number of times that a random match could achieve an equivalent or superior match score, where a lower E-value typically denotes a higher confidence level in the motif match. The q-value addresses the risk of inadvertently finding significant matches due to multiple comparisons by adjusting the *p* value for multiple hypothesis testing, thereby controlling the false discovery rate. A lower q-value suggests that the found motif retains statistical significance even after accounting for multiple comparisons. We performed motif finding on 180 human ATAC-seq datasets and 80 mouse ATAC-seq datasets. Table 2 and Additional file 1: Table S4 shows that on 180 human ATAC-seq datasets, MMGAT found 389 motifs with the highest motifs number. Similarly, Additional file 1: Table S5 demonstrates that on 80 mouse ATAC-seq datasets, MMGAT found the highest number of motifs with 356. The *p* values of motifs found by each model suggest that MMGAT finds higher quality motifs compared to the existing tools. It is worth noting that 'MMGraph + GAT' performs better compared to MMGraph and weaker compared to MMGAT on the human and mouse ATAC-seq datasets. To evaluate the model's running efficiency, this study conducted tests on four differently scaled datasets on an Ubuntu server equipped with 80 cpus kernels and a RTX 2080 GPU. Each dataset was further divided into ten subsets, with the number of positive sequences in each fixed at 10k, 20k, 30k, and 40k, respectively. The performance of each model was evaluated by normalizing its average construction time on the same size dataset. Ultimately, the scalability score was defined as the normalized value of the average build time of these models on four different sized datasets. Figure S2 shows that in scalability, MMGAT has a score of 2.12, which is lower than the scFAN and MMGraph scores but higher than the FactorNet.

**Table 2:** The number of motifs found by the five models on 180 human ATAC-seq datasets, along with the *p* value for the enrichment of the found motifs

Models	scFAN	FactorNet	MMGraph	MMGraph + GAT	MMGAT
Motifs number	348	256	374	383	**389**
$-{{\text{log}}}_{10}\left(p\_value\right)$	3.854	3.759	6.768	7.085	**7.271**

The bold black in table represents the highest value

### Web server application

**Fig. 2:**
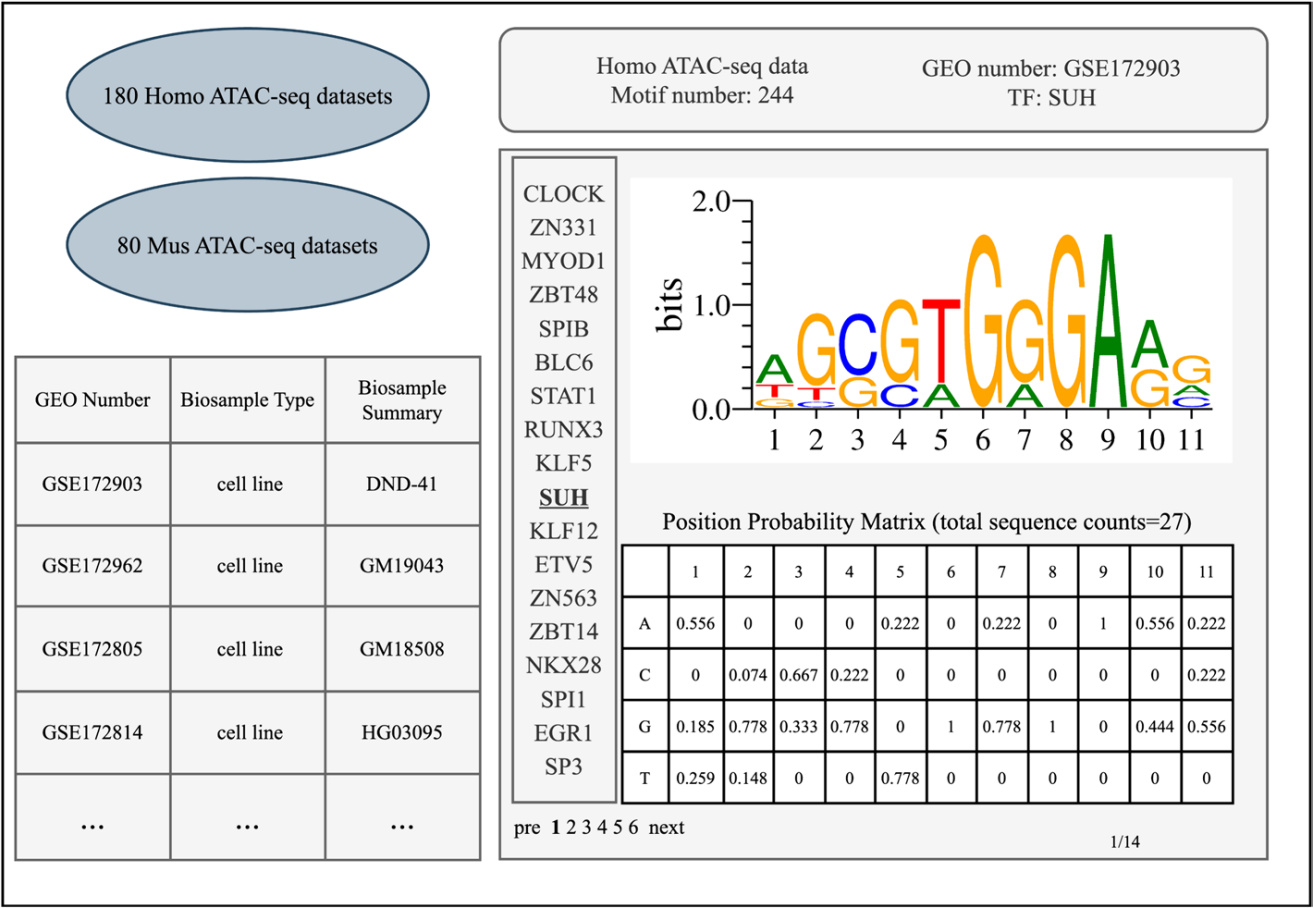
Visualization page of motif finding results on MMGAT-S

While ATAC-seq is generally considered straightforward and robust, there is a limited availability of bioinformatics analysis tools and servers specifically developed for ATAC-seq data [6]. Therefore, we have developed MMGAT-S, a user-friendly and specialized platform for motif-related analyses. MMGAT-S uses Vue3 as the front end and Node.js as the back end [24, 25]. MMGAT-S incorporates the MMGAT tool, enabling users to conduct the TFBS prediction and motif finding by uploading an ATAC-seq dataset. MMGAT-S also offers a visual interface that displays MMGAT's motif finding results from 180 human ATAC-seq datasets and 80 mouse ATAC-seq datasets (Fig. 2). Additionally, MMGAT-S integrates various pre-existing tools, such as AME and GOMo, enabling users to effortlessly perform downstream analyses on the found motifs [15, 16].

## Discussion and conclusion

In this study, we propose an improved MMGAT method for finding multiple ATAC-seq motifs based on the original MMGraph method. MMGAT is a GAT model, uses the attention mechanism to learn k-mer and sequence embeddings, and predicts TFBSs. ATAC-seq motifs are then found using attention coefficients and coexisting probabilities. We conducted experiments on 180 human and 80 mouse ATAC-seq datasets to validate the effectiveness of our proposed method. In the TFBSs prediction task, MMGAT achieves a satisfactory improvement in all six metrics compared to other methods. Especially on the recall metrics, it achieved 2.56% and 6.51% improvement on human and mouse ATAC-seq datasets, respectively. In the comparative experiments, both “MMGraph + GL1” and “MMGraph + GL2” demonstrated performance improvements over MMGraph on most metrics, illustrating that introducing attention mechanisms at both the first and second layers can effectively learn node embeddings in heterogeneous graphs, thereby enhancing prediction accuracy. Howerver, the similar performance of “MMGraph + GL1” and “MMGraph + GL2” makes it challenging to determine the layer at which the addition of the attention mechanism plays a more crucial role. Furthermore, although both variants outperform the original MMGraph model in TFBS prediction performance, they still do not reach the level of MMGAT due to the incomplete application of the attention mechanism. Our results suggest that MMGAT can better represent k-mer and sequence embedding by introducing an attention mechanism in GNN, thus playing an advantage in TFBS prediction. In finding ATAC-seq motifs, MMGAT utilizes the attention coefficients between sequence nodes and k-mer nodes as well as the coexisting probabilities of k-mers to find multiple motifs. We used *p* values to assess the quality of ATAC-seq motifs found by all models, and the results show that MMGAT is the best model for finding multiple ATAC-seq motifs. In the ablation experiments, 'MMGraph + GAT' outperforms MMGraph, indicating that updating the GNN model to GAT not only improves the performance of TFBS prediction, but also brings some positive enhancement to motif finding. In addition, MMGAT outperforms 'MMGraph + GAT', verifying that our approach of using the attention coefficients to replace MI to find k-mer seeds is effective. In terms of scalability, the scores for MMGraph and MMGAT were 2.48 and 2.12, respectively, lower than scFAN but higher than FactorNet. This indicates that both MMGraph and MMGAT require a significant amount of time to construct heterogeneous graphs when dealing with large-scale datasets. Additionally, the lower scalability score of MMGAT compared to MMGraph suggests that the introduction of an attention mechanism, which allocates different weights to the interactions between each node and its neighbors, necessitates additional time and computational resources to determine these weights.

MMGAT also has some limitations. First, MMGAT requires high hardware resources and may face memory limitations when running on large graphs. Second, although MMGAT also achieved the best performance on the mouse ATAC-seq dataset, there is still a lot of room for improvement compared to its performance on the human ATAC-seq dataset. This performance difference mainly stems from the fact that the human ATAC-seq dataset is much larger than the mouse dataset, which prompted us to focus our resources on model optimization on the human dataset. We then attempted to apply the optimized model directly to the mouse dataset, rather than optimizing it specifically for the mouse dataset. This resulted in MMGAT's performance on the mouse dataset being slightly lower than its performance on the human dataset. This suggests that there may be differences in the adaptability of MMGAT to different biological data, and thus further optimization and adaptation is needed to improve generalization capabilities across different species. Our forthcoming efforts will be dedicated to addressing these challenges. Finally, the data preprocessing strategy and heterogeneous map construction method of our proposed MMGAT method are designed for basic ATAC-seq data. In addition to the basic ATAC-seq data, there are common ATAC-seq data types such as scATAC-seq data, snATAC-seq data, and so on. Through extensive comparative analysis, we observed that applying the MMGAT model to these alternative ATAC-seq data types proves challenging. This observation has set the direction for our future research endeavors, aiming to enhance the model's applicability across different types of ATAC-seq datasets.

It is to be expected that the rapid development in the field of GNN opens up new possibilities to further improve model performance. In particular, recent GNN approaches, such as Graph Transformer [26], may provide new perspectives and technical paths to improve and enhance MMGAT due to their advanced performance in processing graph-structured data.

Transcription factors play a crucial role in the gene transcription process by binding to specific TFBSs to either promote or inhibit gene expression [11]. These TFBSs are significant in the pathogenesis of diseases [1]. ATAC-seq data can detect open DNA regions across the genome, allowing the finding of multiple TFBSs through its analysis. By examining ATAC-seq data, we can explore the role of TFBSs in disease development and gene regulation. Therefore, this study provides a potential model for researching TFBSs. Additionally, we developed a web server based on MMGAT, named MMGAT-S. MMGAT-S provides experimental biologists with a user-friendly interactive exploration tool for finding ATAC-seq motifs and conducting downstream analyses on the found motifs. MMGAT-S also provides visualized results of motif finding from MMGAT. This valuable resource empowers researchers to perform efficient and accurate motif finding without the need for programming expertise. In summary, this study presents a practical GAT framework and a user-friendly web server for finding ATAC-seq motifs.

### Supplementary Information

**Additional file 1.** Data and Supplementary Experimental Results.

## Data Availability

The datasets analyzed during the current study are available in the ENCODE repository, https://www.encodeproject.org/. MMGAT-S is freely available at https://www.mmgraphws.com/MMGAT-S/, and the open-source code of MMGAT can be found at https://github.com/xiaotianr/MMGAT. The data underlying this article are available in its online supplementary material.

## Abbreviations

TFs: Transcription factors
ChIP-seq: Chromatin Immunoprecipitation Sequencing
ATAC-seq: Assay for Transposase-Accessible Chromatin using sequencing
TFBSs: Transcription factor binding sites
CNN: Convolutional neural network
GNN: Graph neural network
GAT: Graph attention network
PPMs: Position probability matrices
GO: Gene Ontology
MI: Mutual information
bed narrowPeak: Browser Extensible Data NarrowPeak
bam: Binary Alignment/Map
bp: Base pairs
GEO: Gene Expression Omnibus
