# Supplementary material for: MMGAT: a graph attention network framework for ATAC-seq motifs finding
Source: BMC Bioinformatics. 2024 Apr 20;25:158. doi: 10.1186/s12859-024-05774-x (PMC11031952; doi:10.1186/s12859-024-05774-x)
Supplement: Supplementary file 1 — Additional file 1. Data and Supplementary Experimental Results. [file 12859_2024_5774_MOESM1_ESM.docx]

**Table S1.** The Gene Expression Omnibus (GEO) number of 180 Human ATAC-seq datasets.

| GSE172888 | GSE169771 | GSE172929 | GSE172796 |
| --- | --- | --- | --- |
| GSE187543 | GSE172806 | GSE172790 | GSE172623 |
| GSE172635 | GSE172867 | GSE187737 | GSE187134 |
| GSE94216 | GSE172884 | GSE94170 | GSE215678 |
| GSE172595 | GSE187462 | GSE94179 | GSE215524 |
| GSE94187 | GSE215562 | GSE94175 | GSE172879 |
| GSE172558 | GSE172913 | GSE215522 | GSE187636 |
| GSE172943 | GSE187046 | GSE172575 | GSE215478 |
| GSE172761 | GSE215642 | GSE215477 | GSE172735 |
| GSE172925 | GSE172883 | GSE94195 | GSE215703 |
| GSE215704 | GSE215668 | GSE172903 | GSE215727 |
| GSE172963 | GSE172714 | GSE170227 | GSE215609 |
| GSE172962 | GSE215720 | GSE187257 | GSE215700 |
| GSE172988 | GSE172766 | GSE172839 | GSE215649 |
| GSE215650 | GSE215690 | GSE215517 | GSE172773 |
| GSE215492 | GSE173041 | GSE187695 | GSE215621 |
| GSE187084 | GSE172991 | GSE215583 | GSE170848 |
| GSE187830 | GSE172981 | GSE169891 | GSE170073 |
| GSE172864 | GSE172792 | GSE170578 | GSE170909 |
| GSE169809 | GSE170821 | GSE172802 | GSE170282 |
| GSE170518 | GSE169772 | GSE172685 | GSE170899 |
| GSE187043 | GSE187312 | GSE187174 | GSE172711 |
| GSE170321 | GSE170862 | GSE188147 | GSE187937 |
| GSE187710 | GSE170869 | GSE139763 | GSE172647 |
| GSE187915 | GSE170968 | GSE188076 | GSE187195 |
| GSE187933 | GSE187646 | GSE188031 | GSE170014 |
| GSE187858 | GSE172638 | GSE172841 | GSE173023 |
| GSE172620 | GSE173043 | GSE169955 | GSE172939 |
| GSE172760 | GSE172592 | GSE172949 | GSE172803 |
| GSE172751 | GSE172634 | GSE172699 | GSE172979 |
| GSE172793 | GSE172772 | GSE172700 | GSE114202 |
| GSE172626 | GSE172598 | GSE172679 | GSE172904 |
| GSE172986 | GSE172553 | GSE170824 | GSE173027 |
| GSE172970 | GSE172982 | GSE172833 | GSE169929 |
| GSE170337 | GSE172894 | GSE172878 | GSE169767 |
| GSE172603 | GSE172869 | GSE172710 | GSE172975 |
| GSE170918 | GSE172674 | GSE172797 | GSE173002 |
| GSE172593 | GSE172731 | GSE172702 | GSE172934 |
| GSE170012 | GSE172966 | GSE172646 | GSE172546 |
| GSE172722 | GSE172814 | GSE172518 | GSE172538 |
| GSE170378 | GSE172945 | GSE170251 | GSE172826 |
| GSE172959 | GSE172990 | GSE170214 | GSE172954 |
| GSE172788 | GSE172727 | GSE172775 | GSE172977 |
| GSE172886 | GSE172976 | GSE170245 | GSE172719 |
| GSE173067 | GSE172846 | GSE172952 | GSE172805 |

**Table S2.** The GEO number of 80 Mouse ATAC-seq datasets. The presence of a NULL is due to the lack of a GEO number for this ATAC-seq dataset in the ENCODE project, its ENCODE Experiment ID is ENCSR351QUO.

| GSE172562 | GSE172837 | GSE172670 | GSE172627 |
| --- | --- | --- | --- |
| GSE172630 | GSE172917 | GSE172871 | GSE172845 |
| GSE172876 | GSE172676 | GSE172998 | GSE172763 |
| GSE172625 | GSE172663 | GSE172911 | GSE172703 |
| GSE172784 | GSE172664 | GSE172935 | GSE172812 |
| GSE172572 | GSE172861 | GSE172740 | GSE173061 |
| GSE172659 | GSE173047 | GSE172782 | GSE94211 |
| GSE94181 | GSE173052 | GSE172866 | GSE172900 |
| GSE172597 | GSE172933 | GSE172582 | GSE172767 |
| GSE172576 | GSE173018 | GSE172657 | GSE172738 |
| GSE172667 | GSE173015 | GSE172547 | GSE172561 |
| GSE172723 | GSE172531 | GSE172887 | GSE172616 |
| GSE172969 | ENCSR351QUO | GSE173028 | GSE172554 |
| GSE172688 | GSE173036 | GSE172769 | GSE172843 |
| GSE172821 | GSE172658 | GSE173064 | GSE94221 |
| GSE173037 | GSE172610 | GSE172997 | GSE172770 |
| GSE172745 | GSE173049 | GSE172713 | GSE172818 |
| GSE172698 | GSE94226 | GSE172853 | GSE94212 |
| GSE172912 | GSE172800 | GSE173025 | GSE172535 |
| GSE94167 | GSE172668 | GSE172744 | GSE172852 |

**Table S3.** Mean and standard deviation of precision, recall, F1_score, ACC, AUC, and PRC scores of six models on 80 mouse ATAC-seq datasets.

| Models | scFAN | Factornet | MMGraph | MMGraph+GL1 | MMGraph+GL2 | MMGAT |
| --- | --- | --- | --- | --- | --- | --- |
| Precision | 0.736±0.065 | 0.750±0.049 | 0.855±0.044 | 0.861±0.059 | 0.865±0.055 | **0.893±0.041** |
| Recall | 0.680±0.097 | 0.726±0.062 | 0.830±0.057 | 0.872±0.060 | 0.866±0.058 | **0.884±0.045** |
| F1_score | 0.649±0.139 | 0.716±0.074 | 0.824±0.063 | 0.871±0.061 | 0.872±0.065 | **0.883±0.046** |
| ACC | 0.680±0.097 | 0.725±0.062 | 0.828±0.058 | 0.871±0.060 | 0.872±0.060 | **0.884±0.046** |
| AUC | 0.795±0.092 | 0.827±0.058 | 0.942±0.040 | 0.947±0.044 | 0.947±0.043 | **0.952±0.026** |
| PRC | 0.803±0.093 | 0.832±0.066 | 0.941±0.043 | 0.944±0.052 | 0.943±0.050 | **0.953±0.025** |


**Table S4.** The E-value and q-value of motifs found by the five models on 180 human ATAC-seq datasets.

| **Models** | **scFAN** | **FactorNet** | **MMGraph** | **MMGraph+GAT** | **MMGAT** |
| --- | --- | --- | --- | --- | --- |
| $-{log}_{10} \left( E\_value \right)$ | 1.251 | 1.156 | 4.165 | 4.482 | **4.668** |
| $-{log}_{10} \left( q\_value \right)$ | 1.831 | 1.768 | 4.290 | 4.623 | **4.870** |

**Table S5.** The number of motifs found by the five models on 80 mouse ATAC-seq datasets, along with the p-value, E-value and q-value for the found motifs.

| **Models** | **scFAN** | **FactorNet** | **MMGraph** | **MMGraph+GAT** | **MMGAT** |
| --- | --- | --- | --- | --- | --- |
| Motifs number | 284 | 199 | 347 | 353 | **356** |
| $-\log_{10} \left( p\_value \right)$ | 3.928 | 3.664 | 6.933 | 7.061 | **7.430** |
| $-{log}_{10} \left( E\_value \right)$ | 1.374 | 1.111 | 4.379 | 4.507 | **4.876** |
| $-{log}_{10} \left( q\_value \right)$ | 1.813 | 1.731 | 4.581 | 4.626 | **4.997** |


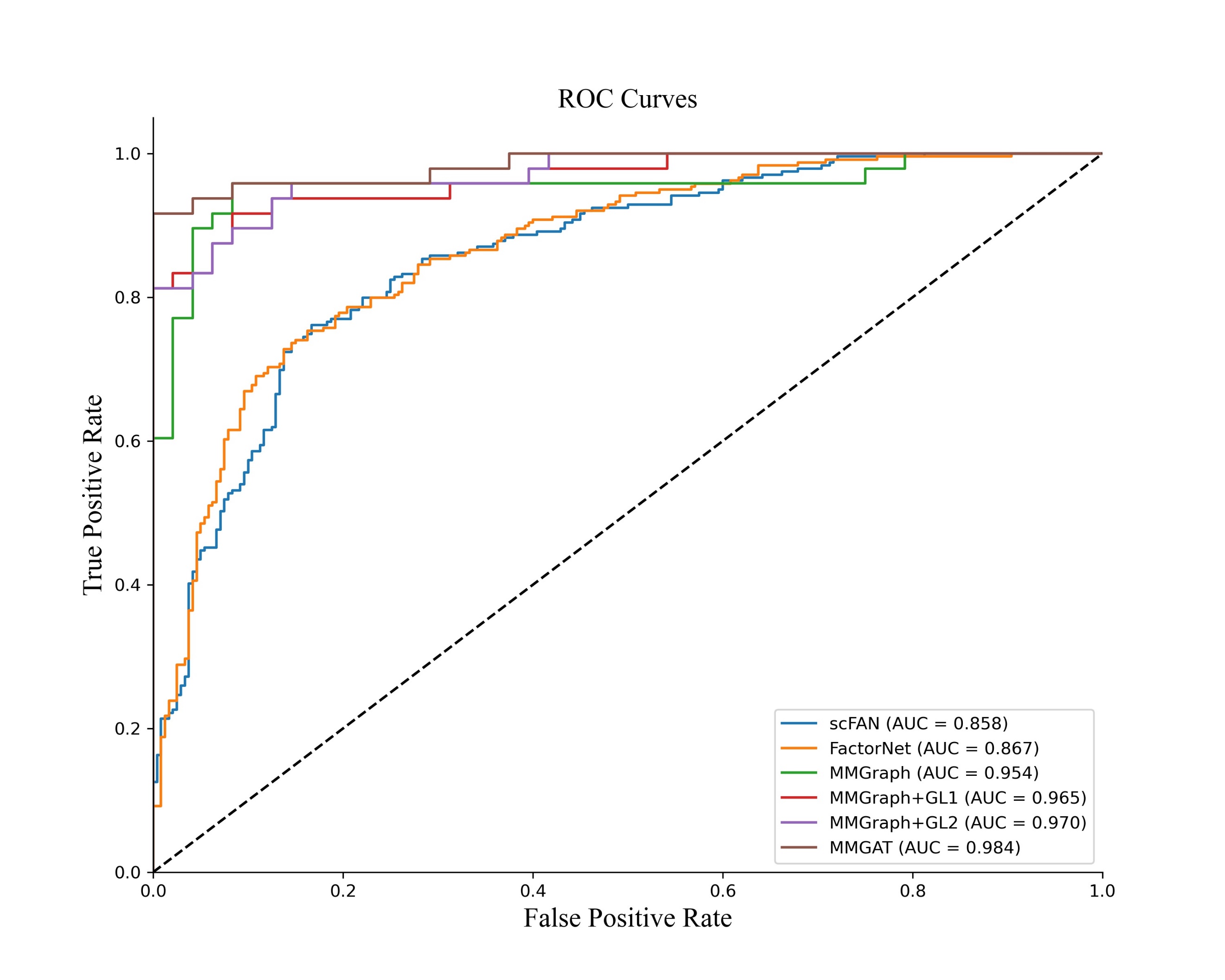


**Figure S1.** The ROC curves of six models.


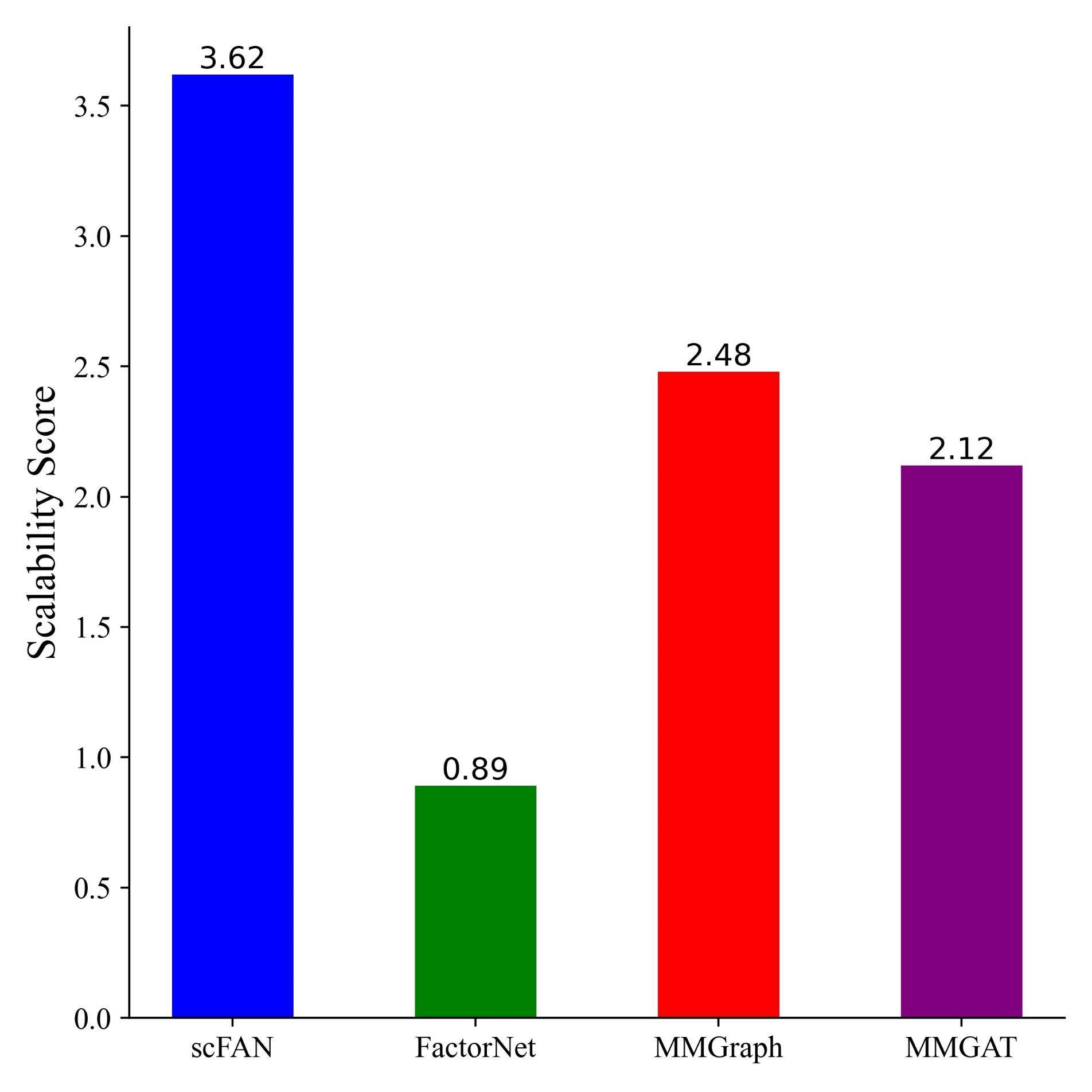


**Figure S2.** The scalability scores of four models.

**Parameter Analysis**

Through parameter sensitivity analysis of the MMGAT model on a validation set of 20 human ATAC-seq datasets for TFBSs prediction, using the average AUC as the evaluation metric, we aimed to explore the impact of parameters $d_{k}$ and dropout rate on the model's performance in predicting TFBSs. The value of $d_{k}$ was selected from the range of {50, 100, 150, 200, 250, 300}. Figure S3 demonstrates that the AUC of the MMGAT model initially increases with the value of $d_{k}$, reaching a peak at $d_{k}$=100, after which further increases in $d_{k}$ result in a decrease in AUC. The dropout rate was explored within {0.1, 0.2, 0.3, 0.4, 0.5, 0.6, 0.7, 0.8, 0.9}, and as shown in Figure S4, the AUC metric exhibits a trend of increasing and then decreasing, reaching its maximum at a dropout rate of 0.3. Based on these findings, the parameters for the MMGAT model have been finalized as $d_{k}=d_{seq}=100$, with a dropout rate of 0.3.


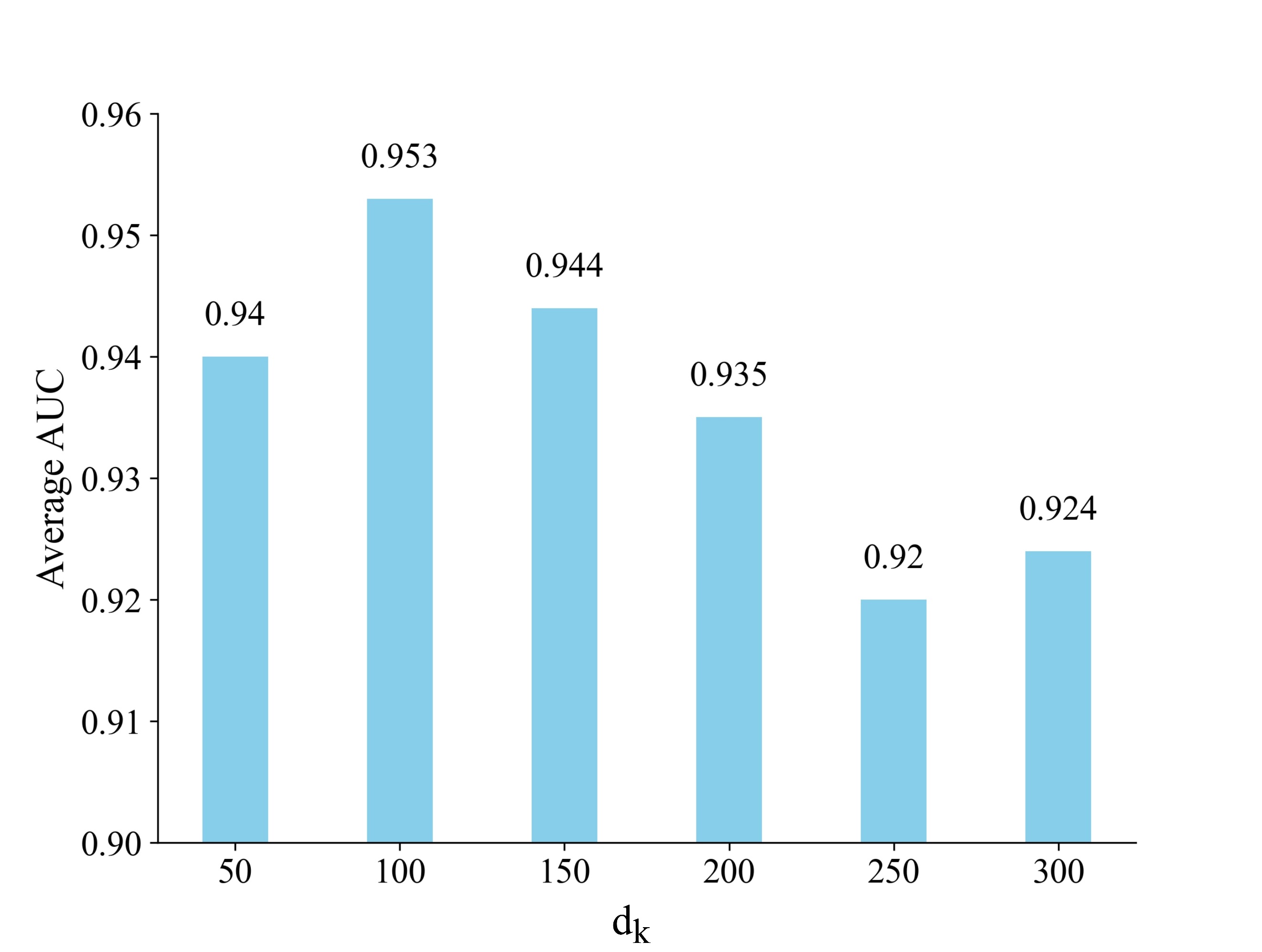


**Figure S3.** Average AUC metric of MMGAT model for different $d_{k}$.


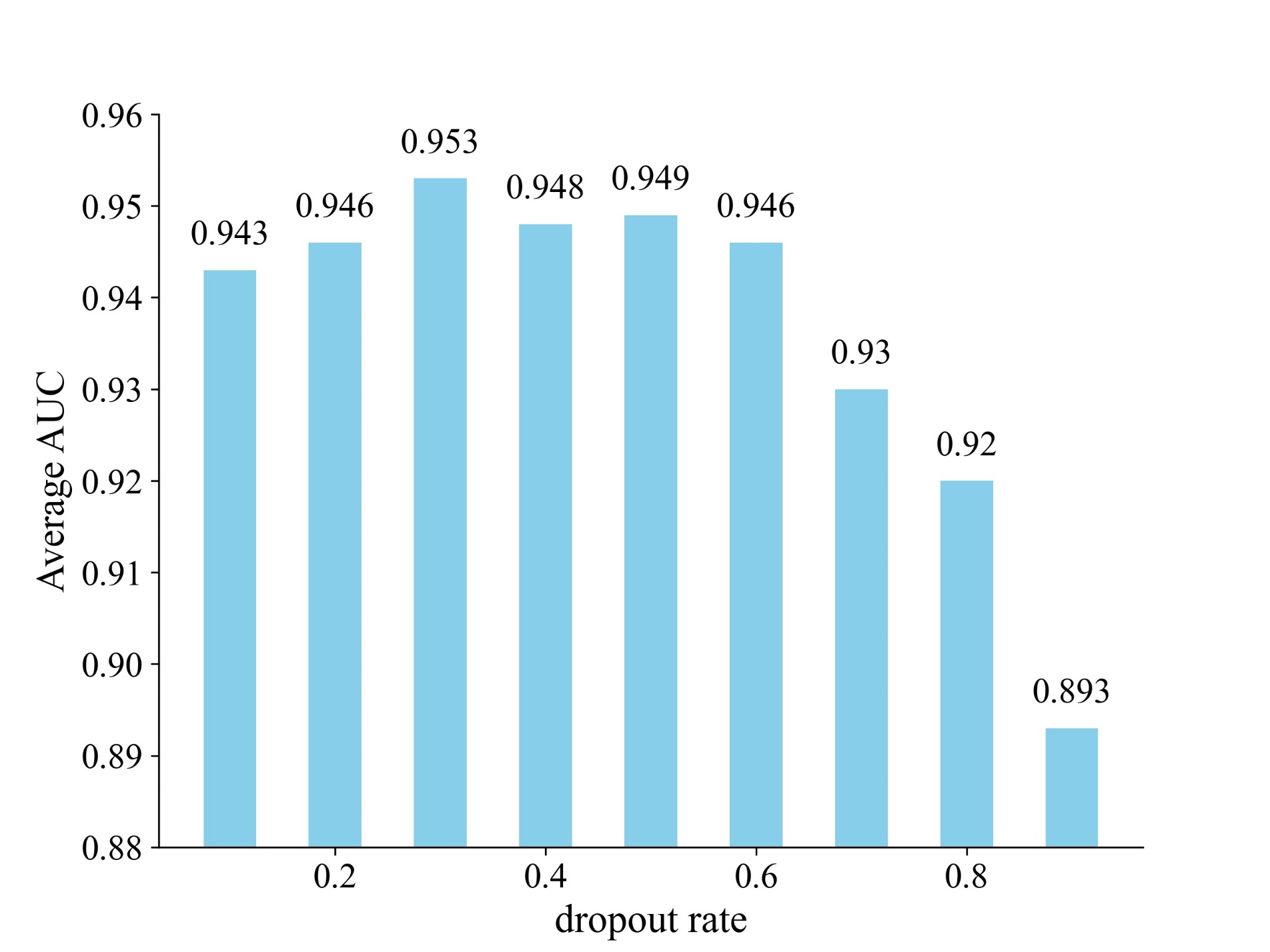


**Figure S4.** Average AUC metric of MMGAT model for different dropout rate.
